# Clown care in the clinical nursing of children: a meta-analysis and systematic review

**DOI:** 10.3389/fped.2024.1324283

**Published:** 2024-03-25

**Authors:** Lina Wang, Jing Zhu, Ting Chen

**Affiliations:** Day Operation Ward, Children’s Hospital of Nanjing Medical University, Nanjing, China

**Keywords:** clown, care, children, anxiety, pain, nursing

## Abstract

**Background:**

Children treated in hospitals often experience high levels of anxiety and pain. The purpose of this meta-analysis was to analyze the effect of clown care in clinical nursing on children and to provide ideas for improving the clinical nursing care provided to children.

**Methods:**

Two authors searched PubMed, Embase, Clinical trials, Cochrane Library, Web of Science, CINAHL, Scopus, China National Knowledge Infrastructure (CNKI), Weipu, and Wanfang databases to identify randomized controlled trials (RCTs) related to clown care for children until 15 September 2023. The quality assessment of the included RCTs and the data extraction were performed by two researchers, and meta-analysis was carried out using RevMan5.4.

**Results:**

A total of 15 RCTs involving 2,252 children were finally included in this meta-analysis. The findings from this meta-analysis revealed that clown care was beneficial in reducing the pain [SMD = −0.96, 95% CI (−1.76, 0.16)], anxiety [SMD = −0.81, 95% CI (−1.16, −0.46)], and crying time [SMD = −1.09, 95% CI (−1.74, −0.44)] of children and the anxiety level of caregivers [SMD = −0.99, 95% CI (−1.95, −0.03)] (all *P*’s < 0.05). No significant publication biases were detected in the synthesized outcomes (all *P*’s > 0.05).

**Conclusions:**

Clown care is helpful in reducing the pain, anxiety, and crying time of children and the anxiety level of caregivers. However, additional high-quality studies with larger sample sizes are warranted to further analyze the role of clown care in clinical practice.

## Introduction

The hospital environment serves as an important stressor for children when seeking a doctor, and the medical and nursing procedures can easily have a negative impact on the experience for children (1). Even minor surgeries or routine treatments can cause pain and anxiety in children. Poor medical experience is not only not conducive to the cure of children's diseases and the development of physical and mental health but also places pressure on their caregivers (2, 3). The psychological and physical development of children is not yet mature, and their psychology and behavior are easily affected by external environmental factors (4). The unfamiliar hospital environment and medical procedures can act as stressors, which can lead to strong stress reactions before an operation and affect the degree of combination of treatment (5). Some studies (6, 7) have shown that most children have serious crying during the perioperative period, which can increase the incidence of postoperative adverse reactions, aggravate the degree of postoperative pain and prolong the postoperative recovery time. Some scholars (8, 9) have proposed that effective nursing intervention in the perioperative period can reduce the stress reaction, improve the degree of coordination, and shorten the recovery time after the operation. Therefore, reducing children's anxiety, pain, and other sad emotions is of great significance to the prognosis of children.

At present, a variety of non-drug intervention methods are used to relieve children's pain and anxiety associated with medical operations, and among these methods, clown care has demonstrated a significant effect. Clown care involves laughter and humor to make patients forget physical pain and mental trauma through, thus speeding up the recovery of patients (10). Clown care is an interdisciplinary intervention that encourages patients to overcome frustration and anxiety by providing the comfort through humor and love to improve their overall condition during hospitalization (11, 12). Clown care is derived from the circus and is used in disease treatment. People who play the role of clowns wear red-nosed faces and strange dramatic costumes. Employing humorous elements like impromptu performance or magic tricks, clown care aims to make patients happy and relaxed to improve their emotional and psychological state and promote their recovery (13, 14). Clown care has been widely used in pediatric wards abroad, and complete clown care systems have even been established in Italy and other countries; however, the development of clown care in many countries is relatively recent, and the effectiveness of this intervention is still controversial. Therefore, in this study, meta-analysis was used to evaluate the intervention and nursing effects of clown care on children to provide reliable references and evidence-based support for clinical child care practices.

## Methods

This systematic review and meta-analysis was performed according to the guidelines of Preferred Reporting Items for Systematic Reviews and Meta-Analyses (PRISMA) (15).

The authors searched the PubMed, Embase, Clinical trails, Cochrane Library, Web of Science, CINAHL, Scopus, China National Knowledge Infrastructure (CNKI), Weipu, and Wanfang databases to identify randomized controlled studies (RCTs) related to clown care for children until 15 September 2023. The English search strategy we used in this study was as follows: (“child*” OR “child*” OR “pediatric*” OR “kids”) AND (“clown” OR “play” OR “Joker”). The computational retrieval adopted the retrieval method that combined topic words with free words, and we also tracked the relevant reviews and references of included RCTs.

The inclusion criteria for this meta-analysis are as follows: the study design had to be an RCT; the subjects had to be children aged ≤18 years without a mental disorder; the RCT compared the effects of clown care vs. routine nursing; and the article reported the corresponding outcome indicators, such as pain, anxiety, and crying time of children anxiety level of caregivers. The literature exclusion criteria for this meta-analysis are as follows: abstracts, conference papers, reviews, and case reports; literature in languages other than Chinese and English; literature reports for which full text could not be obtained or data could not be extracted.

Literature screening and data extraction were carried out by two researchers independently. We removed the weight by Endnote software, then read the title and abstract for literature screening, and finally read the full text for screening to determine the included literature. In the case of disagreements, the two authors engaged in discussions or reached a decision with the help of a third researcher. The data extraction process involved collecting information about the first author, year of publication, country, sample size, characteristics of research objects, intervention measures, evaluation tools, and outcome indicators.

Two researchers used the quality evaluation tools recommended by the Cochrane manual to evaluate the quality of the included RCTs. The evaluation included the following contents: random sequence, allocation hiding schemes, whether to use the blind method, result evaluator blind method, whether the result data were complete, whether the results were selectively reported, and other biases. In the case of differences in quality evaluation, the two researchers negotiated together or sought input from the third researcher to make a decision.

In this meta-analysis, RevMan 5.3 software was used to analyze the data. For continuous variables, standardized mean difference (SMD) was used for statistical analysis, and we calculated the 95% confidence interval (95% CI). Heterogeneity was evaluated using the *I*^2^ value. If *P* < 0.1 and *I*^2^ > 50%, it indicated that the heterogeneity was large, and consequently, the random effects model was used for data analysis. If *P* ≥ 0.1 and *I*^2^ ≤ 50%, it indicated that the studies were homogeneous, and the fixed effects model was used for merging. Publication biases were evaluated using funnel plots and Egger tests. A statistical difference was considered when the *P*-value was less than 0.05.

## Results

**Figure 1:**
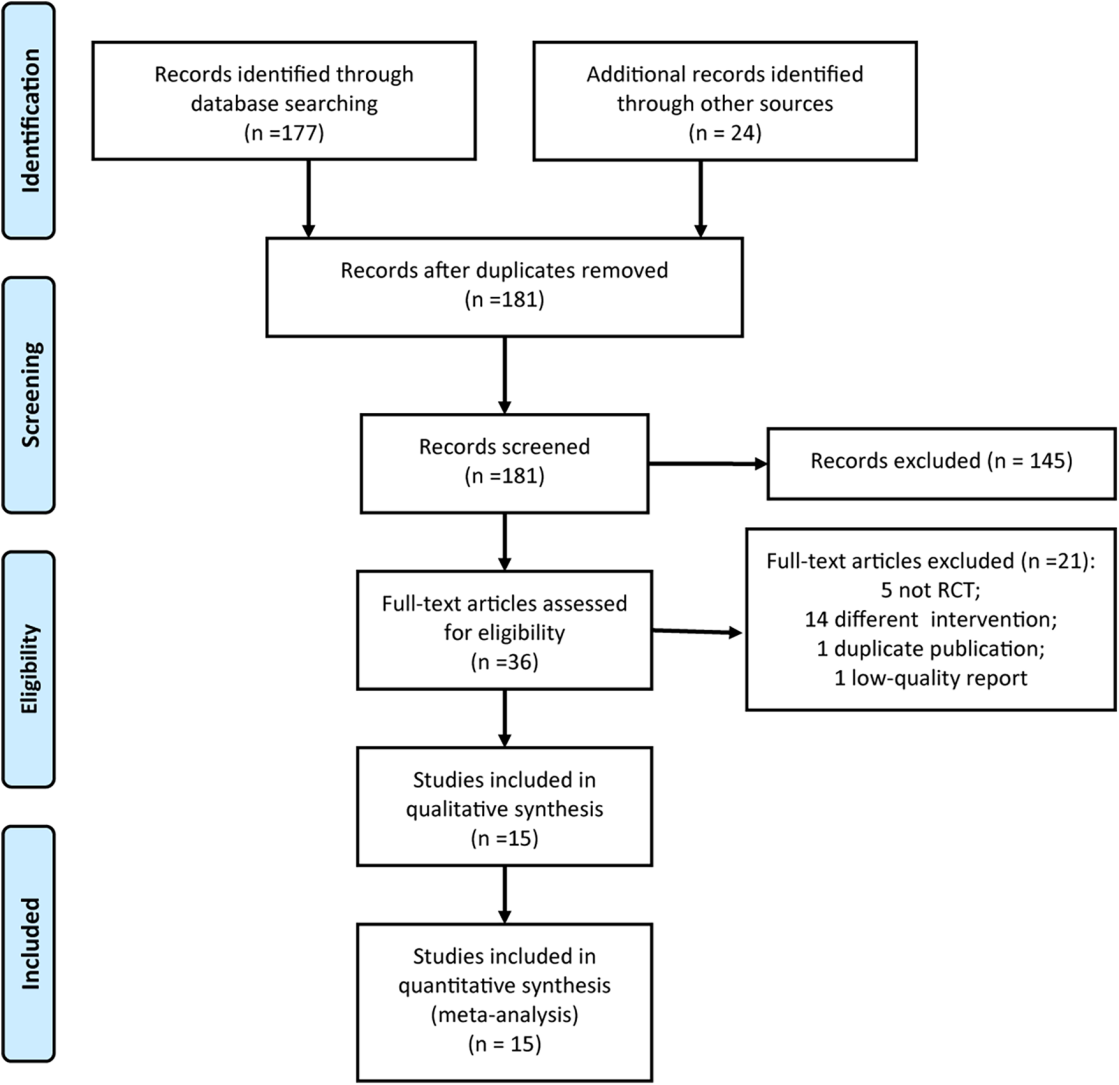
Flowchart of RCT selection.

First, 201 articles were obtained from all the databases; then, we removed the duplicates and screened out the literature according to the inclusion and exclusion criteria. Finally, 15 RCTs (16–30) were included for data analysis. The literature screening process is shown in Figure 1.

In the 15 RCTs (16–30) included, 2,252 children were involved, including 1,150 in the clown care group and 1,102 in the control group. The features of included RCTs are presented in Table 1.

**Table 1:** Characteristics of included RCTs.

Study	Country	Sample size		Age and setting	Interventions		Outcomes
		Clown group	Control group		Clown group	Control group	
Agostini (16)	Italy	25	25	3–12 years, surgery	Interact with the clown doctor for 30 min in the waiting room before the operation	Parent company	STAI
Bertini (17)	Italy	21	22	7–10 years, respiratory diseases	2–5:00 pm interaction with the clown pm during hospitalization	Routine care	Wong–Baker facial expression pain rating scale, length of hospital stay
Cheng (18)	China	143	142	2–12 years, surgery	Situational, behavioral, and psychological clown interventions	Routine care	VAS, crying time
Dionigi (19)	Italy	52	25	2–12 years old, otorhinolaryngology surgery	Two clown doctors interact with children for 30 min, providing pranks, magic, and puppet interventions	Parent company	m-YPAS, STAI
Golan (20)	Israel	21	22	3–8 years, surgery	Interact with the clown for 20–30 min in the waiting area before operation	Parent company	m-YPAS
Goldberg (21)	Israel	45	46	2–17 years, allergic skin test	The child is accompanied by a clown from entering the consultation room to the end of the skin test, more than 15 min	Parent company	M-YPAS, FLACC, VAS, STAI
Heilbrunn (22)	USA	43	29	5–12 years, pediatric emergency room	Interact with clowns for 5 min, including telling jokes, playing with balloons, and listening to music	Routine care	m-YPAS
Meiri (23)	UK	31	33	2–10 years, venipuncture and blood test	The first 10 min of the blood test began to interact with the clown until the end of the puncture	Routine care	VAS, crying time
Newman (24)	Israel	23	22	≥4 years, surgery	Parents’ company, 30–60 min interaction with clown before the operation, anesthesia, and conciliatory interaction after the operation	Parent company	Wong–Baker facial expression pain scale
Rimon (25)	Israel	29	24	2–15 years, emergency venipuncture	Interact with the medical clown for 15 min before venipuncture	Routine care	FPS-R, VAS
Vagnoli (27)	Italy	20	20	5–12 years, surgery	Interaction with the clown 15 min before the operation	Parent company	m-YPAS, STAI
Vagnoli (26)	Italy	25	25	5–12 years, surgery	Interact with the clown for about 15 min before the operation. The clown shows magic tricks, games, puppets, and other performances	Parent company	m-YPAS, STAI
Wolyniez (28)	Israel	26	21	3–16 years, emergency venipuncture	Interact with the medical clown for 15 min before the operation	Routine care	VAS, FPS-R,STAI
Xue (29)	China	46	46	4–12 years, surgery	Situational, behavioral, and psychological clown interventions	Routine care	VAS, HAMA, crying time
Zhang (30)	China	600	600	2–10 years, surgery	Situational, behavioral, and psychological clown interventions	Routine care	m-YPAS, FLACC

STAI, state-trait anxiety inventory; m-YPAS, modified yale preoperative anxiety scale; scale: FLACC, facial expression, lower limb movement, activity, crying, comfort; VAS, visual analog scale; FPS-R, faces pain scale – revised; HAMA, hamilton anxiety scale.

**Figure 2:**
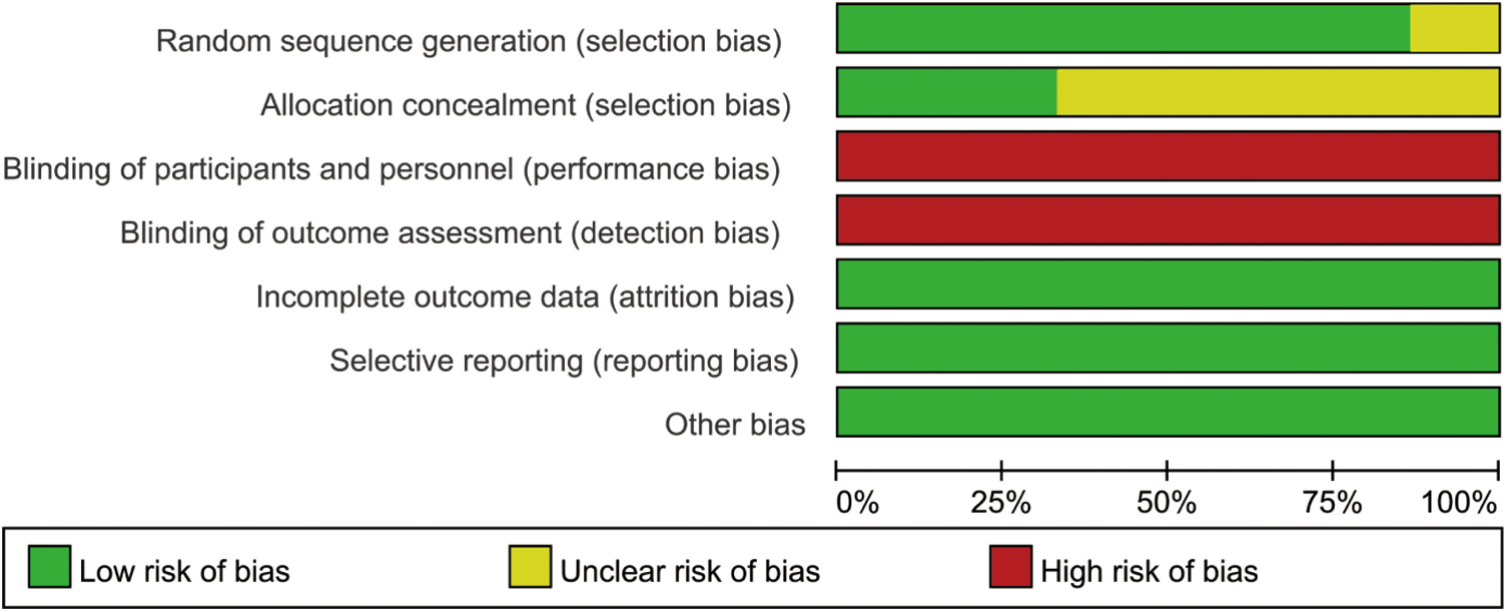
Risk of bias graph.

**Figure 3:**
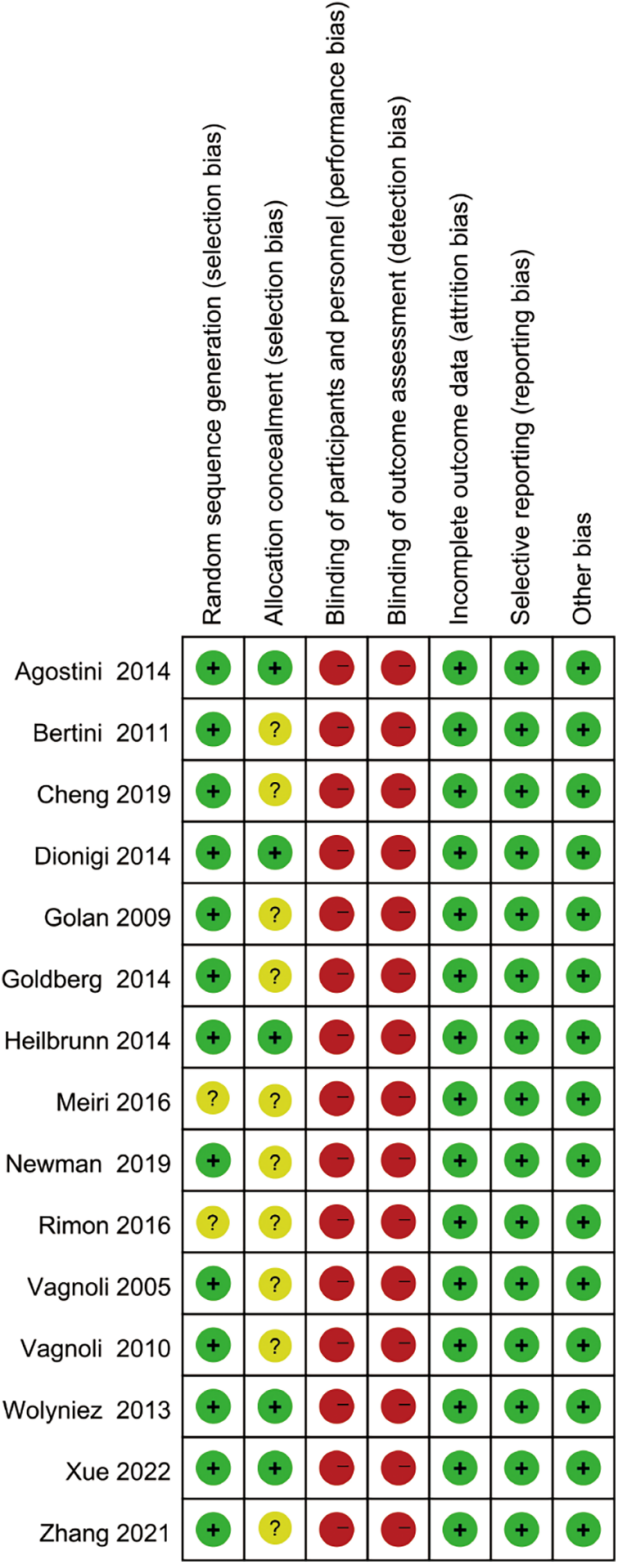
Risk of bias summary.

The methodological quality evaluation included in the study is shown in Figures 2, 3. Of 15 RCTs, 13 RCTs reported specific methods of randomized grouping, while 5 RCTs reported allocation hiding schemes. Because of the nature of clown care, it was difficult to achieve blinding of researchers and participants. All the reported data were complete. There were no selective reports on the results, and no other sources of bias were identified. The baseline data of the two groups of children in each included RCT were comparable.

**Figure 4:**
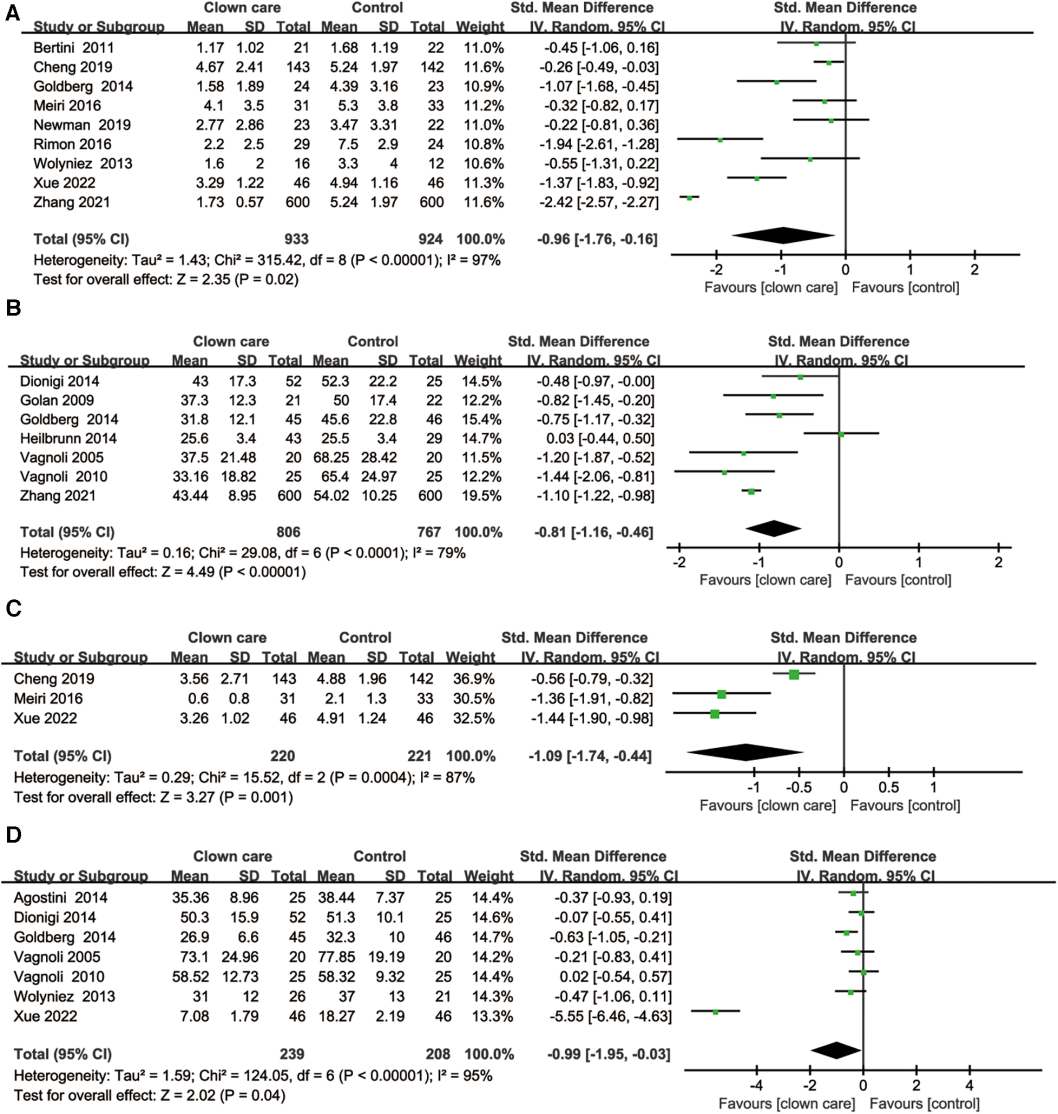
Forest plots of synthesized outcomes. (**A**) Forest plot for children’s pain level. (**B**) Forest plot for children’s anxiety level. (**C**) Forest plot for crying time. (**D**) Forest plot for caregivers’ anxiety level.

*Children's pain level:* Nine RCTs reported the effect of clown care on children's pain scores. There was heterogeneity among the studies (*I*^2 ^= 97%, *P* < 0.001), so the random effects model was selected for analysis. The results showed that clown care could significantly reduce the pain degree of children [SMD = −0.96, 95% CI (−1.76, 0.16), *P* = 0.02], as shown in Figure 4A.

*Children's anxiety level:* Seven RCTs reported the effect of clown care on children's anxiety scores. There was heterogeneity among the studies (*I*^2 ^= 79%, *P* < 0.001), so the random effects model was selected for analysis. The results showed that clown care could significantly reduce the anxiety degree of children [SMD = −0.81, 95% CI (−1.16, −0.46), *P* < 0.001], as shown in Figure 4B.

*Children's crying time:* Three RCTs reported the effect of clown care on children's crying time. There was heterogeneity among the studies (*I*^2 ^= 87%, *P* < 0.001), so the random effects model was selected for analysis. The results showed that clown care could significantly reduce the crying time of children [SMD = −1.09, 95% CI (−1.74, −0.44), *P* = 0.001], as shown in Figure 4C.

*Caregivers’ anxiety level:* Seven RCTs reported the effect of clown care on the anxiety level of caregivers. There was heterogeneity among the studies (*I*^2 ^= 95%, *P* < 0.001), so the random effects model was selected for analysis. The results showed that clown care could significantly reduce the caregivers’ anxiety level [SMD = −0.99, 95% CI (−1.95, −0.03), *P* < 0.001], as shown in Figure 4D.

**Figure 5:**
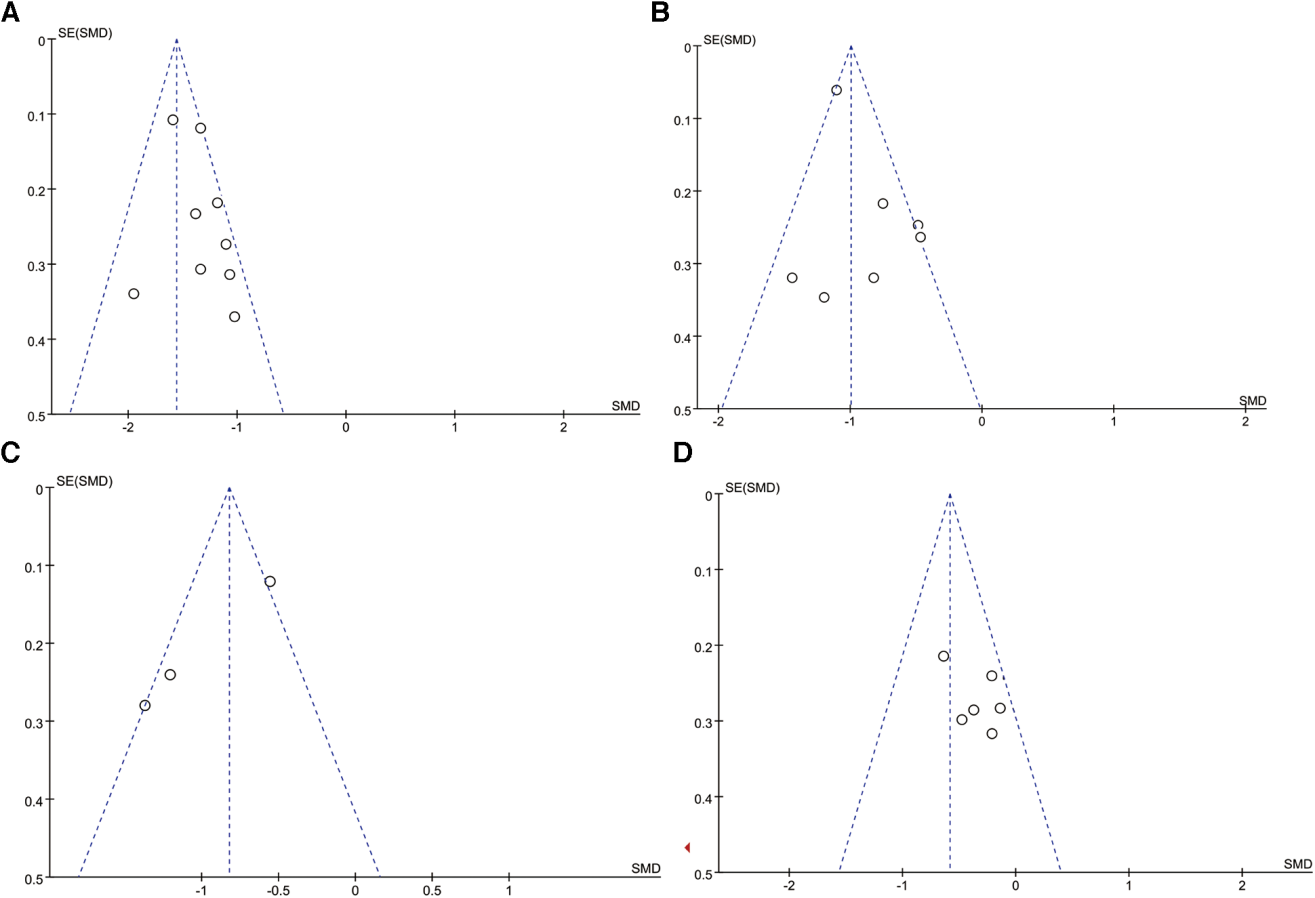
Funnel plots of synthesized outcomes. (**A**) Funnel plot for children’s pain level. (**B**) Funnel plot for children’s anxiety level. (**C**) Funnel plot for crying time. (**D**) Funnel plot for caregivers’ anxiety level.

We conducted sensitivity analysis by removing each outcome index one by one, and the synthesized results showed no significant changes, indicating that the results were relatively stable. As shown in Figure 5, the distribution of points in funnel plots was relatively uniform and symmetrical. The results from Egger’s tests indicated there were no significant publication biases in the synthesized outcomes (all *P*’s > 0.05).

## Discussion

Children represent a special group, susceptible to feelings of fear, resistance, and other sad emotions in the hospital environment; these emotional responses can increase the risk associated with operation and anesthesia, which is not conducive to their postoperative recovery; hence, providing effective nursing interventions for children in the hospital is crucial (31). The psychological needs of children differ from the traditional behavior patterns of medical staff, which leads to poor medical experiences such as the “needle phenomenon” and “white coat phenomenon,” which can aggravate the resistance of children and affect the effectiveness of clinical treatment and nursing care (32, 33). The results of this meta-analysis have shown that clown care is beneficial in reducing the pain degree, anxiety level, and crying time of children and the anxiety level of caregivers, which may be promoted in clinical care.

The results of this meta-analysis show that clown care can reduce children's pain. The results of this meta-analysis differ from those of Sridharan and Sivaramakrishnan (11), which may be related to the different types of literature and sample sizes included in each study. Pain caused by invasive operation procedures is a common experience for children, and effective non-drug interventions can help to reduce pain in children. Compared with drug interventions, non-drug interventions offer advantages such as lower cost, easy operation procedures, and fewer adverse reactions (34). As a non-drug intervention, clown care is based on the theory of positive psychology. According to the age and personality characteristics of children, exaggerated actions are taken to create a relaxed atmosphere. It is worth noting that clown care may vary in its methods and content depending on the age of children involved. On the one hand, pleasant experiences can promote the production of endorphins in the brain, thereby reducing pain (35). In addition, by diverting children's attention, clown care can reduce their attention to pain (36). Therefore, clown care plays a positive role in reducing the pain levels experienced by children.

Our finding that clown care can relieve children's anxiety is consistent with the relevant research results. Due to their immature physical and mental development, children have limited cognitive ability and low self-control, which makes them prone to anxiety in the process of seeking medical treatment (37). Clown nurse Li (38) employs humor to bring joy to children, help them relax, and ease their anxiety. In addition, clown care can shorten the crying time of children. A previous study (39) has reported that clown care can enhance immunity, thus promoting the recovery of children from diseases. Clown care uses exaggerated and humorous movements to amuse children through situational, psychological, and behavioral interventions, which helps to shorten their crying time (40).

Children undergoing treatment in a hospital setting can bring great stress to their parents; the phenomenon of crying and resistance during hospitalization will lead to varying degrees of anxiety, which will affect not only the physical and mental health of the parents but also the rehabilitation and treatment process of children. The medical clown reduces the physical and mental stress reactions of children, relieves the resistance during treatment, reduces the postoperative pain, shortens the crying time, and relieves the anxiety of parents to some extent (41). In addition, when children seek medical treatment, caregivers show excessive anxiety and tension due to a lack of disease-related knowledge and limited experience in providing care (42). Clown care translates professional medical knowledge into everyday terms to increase caregivers’ understanding of disease-related knowledge and alleviate their anxiety levels (10, 35).

There are some limitations in this study that are worth considering. First, the methodological design of RCTs is not rigorous enough to clearly point out the implementation of the random sequence generation method and the hidden distribution. Second, the literature includes the ethnic and cultural background differences, some studies have relatively small sample sizes, the measurement tools used for outcome indicators are inconsistent, and the inconsistency of clown care intervention place, time, and specific implementation content increases the source of heterogeneity. Third, the effects of clown care on children of different ages cannot be evaluated by conducting a subgroup analysis in this meta-analysis because of the limited data; further studies are needed in the future to evaluate the effects of clown care on children across various age groups. Finally, this meta-analysis only includes published Chinese and English literature, and reports with other languages and gray literature are not included, which may bias the results.

## Conclusions

In conclusion, the results of this study show that compared with routine nursing, clown care can reduce children's pain, relieve their anxiety, shorten their crying time, and improve the anxiety level of caregivers. However, some young children might be frightened by clowns and become tearful or anxious. Clowns should be trained to be attentive to and aware of such children and refer to staff and parents to help reduce anxiety until the specific child becomes comfortable with the clowns. At present, there is little high-quality literature on the intervention effect of clown care on children and caregivers. It is suggested that the application field of clown care should be broadened in the future, and more high-quality RCTs should be carried out to further analyze the clinical nursing effect of clown care to provide reliable evidence for clinical treatment and nursing care.
